# Machine Learning-Based Prediction of Long-Term Mortality in STEMI Patients Using Clinical, Laboratory, and Inflammatory–Metabolic Indices

**DOI:** 10.3390/jcm15051800

**Published:** 2026-02-27

**Authors:** Gökhan Keskin, Abdulkadir Çakmak, Mehmet Uğur Çalışkan

**Affiliations:** Department of Cardiology, Faculty of Medicine, Amasya University, Amasya 05200, Türkiye; gokhan.keskin@amasya.edu.tr (G.K.); ugurkobian@gmail.com (M.U.Ç.)

**Keywords:** STEMI, machine learning, XGBoost, mortality, inflammatory indices

## Abstract

**Background:** This study aims to compare the performance of machine learning (ML) models developed to predict long-term mortality risk in patients with ST-segment elevation myocardial infarction (STEMI) undergoing primary percutaneous coronary intervention (pPCI) and to investigate the prognostic value of novel inflammatory–metabolic indices. **Methods:** In this retrospective study, 329 consecutive STEMI patients who underwent pPCI (292 survivors, 37 deaths) were included. Five ML algorithms—Logistic Regression (LR), Random Forest (RF), Extreme Gradient Boosting (XGBoost), Support Vector Machines (SVM), and Artificial Neural Networks (ANN)—were developed for mortality prediction. Model performance was evaluated using accuracy, sensitivity, specificity, and the area under the receiver operating characteristic (ROC) curve (AUC). SHAP (Shapley Additive exPlanations) analysis was used to interpret model decision mechanisms. **Results:** The mortality group had significantly higher door-to-balloon time (DTBT), Systemic Inflammatory Response Index (SIRI), pan-immune-inflammation value (PIV), whereas body mass index (BMI), Prognostic Nutritional Index (PNI), and Advanced Lung Cancer Inflammation Index (ALI) values were significantly lower (*p* < 0.001). Among the ML models, the XGBoost algorithm achieved the best performance, with 98.99% accuracy, a ROC-AUC of 0.999, and 100% sensitivity, correctly identifying all mortality cases. SHAP analysis identified DTBT, albumin level, and ALI score as the strongest predictors of mortality, in that order. **Conclusions:** The XGBoost algorithm provides high accuracy and reliability for predicting long-term mortality in STEMI patients. Beyond DTBT, integrating novel indices—especially ALI and TyG—into ML models may serve as a powerful clinical tool for early identification of high-risk patients and improved risk stratification.

## 1. Introduction

Cardiovascular diseases (CVDs) remain the leading cause of morbidity and mortality worldwide, and ST-segment elevation myocardial infarction (STEMI) represents one of the most lethal clinical manifestations within this spectrum [1]. Despite substantial advances in primary percutaneous coronary intervention (pPCI) and pharmacological therapies, short- and long-term mortality rates in STEMI patients still range between 5% and 10%, underscoring the ongoing critical importance of early risk stratification [2]. Although conventional risk scores such as the thrombolysis in myocardial infarction (TIMI) score and the Global Registry of Acute Coronary Events (GRACE) score are widely used to predict mortality risk, these models typically rely on a limited number of variables and may be insufficient to fully capture the complex pathophysiological status of patients [3,4]. Therefore, there is a need for more comprehensive predictive models incorporating novel, next-generation biomarkers to enable clinicians to identify high-risk patients with greater precision.

It is well established that inflammation plays a pivotal role in the pathogenesis of atherosclerosis and plaque rupture, and that a high inflammatory burden is associated with poor prognosis [5,6]. In this context, biomarkers derived from complete blood count parameters—such as the neutrophil-to-lymphocyte ratio (NLR) and the more contemporary systemic immune-inflammation index (SII)—have been reported in the literature as strong predictors of adverse cardiovascular outcomes in STEMI patients [7,8]. In addition to inflammatory markers, metabolic and nutritional parameters reflecting prognosis—such as the triglyceride–glucose index (TyG), an indicator of insulin resistance, and the C-reactive protein (CRP)/albumin ratio (CAR)—have also been shown to have significant prognostic value [9,10]. However, analyzing a large number of variables that represent diverse physiological pathways (inflammation, metabolism, renal function, etc.) within a single model using traditional linear regression methods introduces notable statistical challenges.

The complexity of medical data and the presence of non-linear relationships among variables necessitate the use of more advanced analytical tools beyond traditional statistical methods [11]. Machine learning (ML) algorithms, a subfield of artificial intelligence (AI), have shown promising results in cardiology due to their superior ability to process high-dimensional data and detect latent patterns among variables [12,13,14,15,16,17,18]. ML-based models not only improve predictive accuracy but also have the potential to inform clinicians about the relative contribution of each parameter to risk.

In this study, we aimed to comprehensively evaluate the prognostic value of multiple inflammatory and metabolic indices (NLR, SII, Systemic Inflammatory Response Index (SIRI), pan-immune-inflammation value (PIV), CAR, TyG, atherogenic index of plasma (AIP), Prognostic Nutritional Index (PNI), and Advanced Lung Cancer Inflammation Index (ALI)) for long-term (24-month) mortality in hospitalized patients diagnosed with STEMI. The study was conducted on a multidimensional dataset integrating these indices with patients’ demographic characteristics, comorbidities (e.g., diabetes, hypertension (HT), chronic obstructive pulmonary disease (COPD), renal failure), laboratory findings (e.g., lipid profile, renal and liver function tests), and procedural parameters (e.g., door-to-balloon time (DTBT), number of diseased vessels, left ventricular ejection fraction (LVEF)). Within this framework, several ML algorithms—including Logistic Regression (LR), Random Forest (RF), Extreme Gradient Boosting (XGBoost), Support Vector Machines (SVM), and Artificial Neural Networks (ANN)—were implemented to build a high-performance prognostic model. The key novelty of this study lies in combining multiple pan-inflammatory and metabolic biomarkers rather than focusing on a single inflammatory marker, and in enhancing clinical applicability by making the model’s decision mechanism explainable using SHAP (SHapley Additive Explanations).

## 2. Materials and Methods

This study was conducted using retrospective clinical data obtained from patients diagnosed with STEMI who presented to the Emergency Department of Amasya University Faculty of Medicine and were subsequently hospitalized, monitored, and treated in the Department of Cardiology. The primary aim of the study was to develop and evaluate ML–based prognostic models capable of predicting mortality in STEMI patients. The dataset comprised multidimensional clinical variables, including demographic data (age, sex, body mass index (BMI)), comorbidities (diabetes mellitus (DM), HT, COPD, chronic kidney disease (CKD), atrial fibrillation (AF), and history of cerebrovascular accident (CVA)), cardiac and procedural parameters (DTBT, number of diseased vessels, and LVEF), and laboratory markers (complete blood count (CBC), glucose, creatinine, C reactive protein (CRP), hemoglobin A1c (HbA1c), lipid profile, and liver function tests). In addition, composite indices reflecting inflammatory and metabolic processes—NLR, SII, SIRI, PIV, CAR, TyG, AIP, PNI, and ALI—were calculated for each patient.

### 2.1. Study Population

This retrospective observational study included adult individuals aged ≥18 years with a confirmed diagnosis of STEMI who were followed and treated in the Department of Cardiology at Amasya University Faculty of Medicine between 10 January 2023 and 18 July 2023. The study was approved by the Amasya University Non-Interventional Clinical Research Ethics Committee (Decision No: 2025/250; approval date: 12 December 2025). Due to the retrospective nature of the study, the requirement for obtaining informed consent from patients was waived by Amasya University Rectorate Non-Interventional Clinical Research Ethics Committee.

Following ethical approval, demographic, clinical, laboratory, and angiographic data were retrospectively reviewed using the hospital information system. The diagnosis of STEMI was confirmed by ST-segment elevation on a 12-lead ECG, typical chest pain and/or elevated cardiac troponin levels, and was supported by coronary angiography findings in all patients.

Inclusion criteria were adult patients with a confirmed STEMI diagnosis who underwent pPCI and had complete clinical and laboratory data. Patients with acute infection, active malignancy, chronic inflammatory disease, advanced liver failure, hematologic disorders, or a history of immunosuppressive therapy were excluded. Cases with missing laboratory records, insufficient angiographic data, or lacking 24-month mortality follow-up were also excluded from the analysis. A total of 1500 patient records with ACS were screened. Of these, 1171 patients were excluded based on the predefined criteria, including missing laboratory data (*n* = 400), active malignancy (*n* = 50), chronic inflammatory disease (*n* = 100), insufficient angiographic data (*n* = 621), and other exclusion conditions as described above. After applying strict inclusion and exclusion criteria, 329 patients with STEMI were included in the final study cohort. Mortality was defined as long-term all-cause mortality occurring during a mean 24-month follow-up period. Follow-up mortality data were obtained through verification and confirmation via the patient’s hospital records and the Central Population Management System (MERNIS-TÜRKİYE), where deaths are registered in our country.

In the study, all patients without AF received ticagrelor plus acetylsalicylic acid (ASA) therapy for one year, whereas patients with AF were treated with ASA for one week and clopidogrel plus a direct oral anticoagulant for one year.

**Figure 1:**
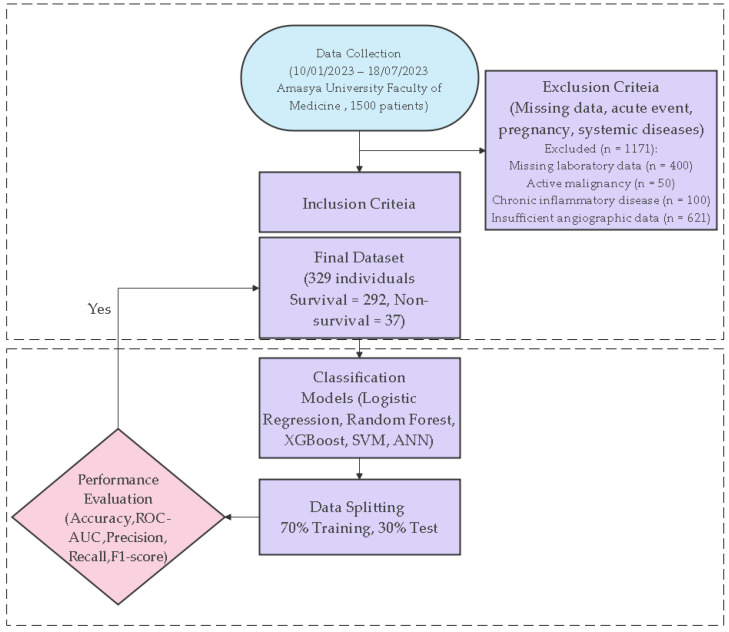
Flowchart of the study workflow.

The methodological workflow of the study—from data collection to model development and performance evaluation—is systematically summarized in Figure 1. This flowchart comprehensively reflects all stages of the research, including patient selection, data preprocessing, modeling, and performance evaluation.

### 2.2. Clinical and Procedural Parameters

Clinical and procedural parameters of STEMI patients included length of hospital stay, DTBT, vascular involvement score, and LVEF. Length of hospital stay reflects clinical recovery time and the risk of developing complications, whereas DTBT represents reperfusion success and treatment effectiveness. The vascular involvement score was determined according to the presence of severe obstructive coronary artery disease identified on coronary angiography and indicates the overall coronary atherosclerotic burden. Based on the number of major epicardial coronary arteries with significant involvement, the vascular score was classified as follows: Score 1 if one major epicardial vessel was involved, score 2 if two vessels were involved, and score 3 if three vessels were involved. These parameters were analyzed to assess their associations with acute clinical management and long-term mortality in STEMI patients.

### 2.3. Laboratory Parameters

Laboratory findings at presentation were evaluated as key biomarkers providing information on systemic inflammatory response, metabolic status, and cardiac function. All laboratory variables were examined in three main groups:

CBC: White blood cell count (WBC), hemoglobin (HGB), platelet count (PLT), neutrophil, lymphocyte, and monocyte values were analyzed. These parameters were used to assess inflammatory activity and hematological balance. In particular, the NLR is an important indicator reflecting systemic inflammation [19].

Metabolic–Biochemical Profile: Glucose, creatinine, estimated glomerular filtration rate (eGFR), liver enzymes (alanine aminotransferase, aspartate aminotransferase, alkaline phosphatase (AST, ALT, ALP)), uric acid, CRP, troponin, and albumin levels were evaluated. These parameters were analyzed to assess metabolic status, renal and hepatic function, inflammatory burden, and myocardial injury.

Lipid Profile: Lipid profile (total cholesterol (TC), low-density lipoprotein cholesterol (LDL-C), high-density lipoprotein cholesterol (HDL-C), triglycerides (TGs)) and HbA1c levels were measured. These markers were used to evaluate cardiometabolic risk and the severity of atherosclerotic processes.

All laboratory data were analyzed to determine the effects of metabolic stress, inflammation, and cardiac injury on mortality in STEMI patients.

### 2.4. Calculated Indices and Ratios

To comprehensively assess systemic inflammatory activity, metabolic status, and nutritional indicators in STEMI patients, several composite indices with established utility in the literature were calculated from laboratory parameters. These indices were evaluated in two main categories: inflammatory indices and metabolic/prognostic indices.

#### 2.4.1. Inflammatory Indices

Hematological ratios reflecting inflammatory response were calculated using the following formulas:

$$\mathrm{NLR}\;=\frac{Neutrophils\;\left(10^{3}/µL\right)}{\mathrm{Lymphocyte}\;\left(10^{3}/µL\right)}$$

$$\mathrm{SII}=\frac{\mathrm{Trombosit}\;\left(10^{3}/µL\right)\;\times \;\mathrm{Neutrophils}}{\mathrm{Lymphocyte}\;\left(10^{3}/µL\right)}$$

$$SIRI=\frac{\mathrm{Neutrophils}\;\left(10^{3}/µL\right)\;\times \;\mathrm{Monocytes}}{\mathrm{Lymphocyte}\;\left(10^{3}/µL\right)}$$

$$PIV=\frac{Platelets\;\left(10^{3}/µL\right)\;\times \;\mathrm{Neutrophils}\;\left(10^{3}/µL\right)\;\times \;\mathrm{Monocytes}\;\left(10^{3}/µL\right)}{\mathrm{Lymphocyte}\;\left(10^{3}/µL\right)}$$

These indices quantitatively reflect the level of systemic inflammatory response and were used as potential biomarkers for predicting post-STEMI mortality risk.

#### 2.4.2. Metabolic and Prognostic Indices

Metabolic status and nutritional indices were calculated as follows:

$$\mathrm{CAR}\;=\frac{\mathrm{CRP}\;\left(\mathrm{mg}/L\right)}{\mathrm{Albümin}\;\left(g/L\right)}$$

$$\mathrm{TyG}=\ln\left(\frac{Triglycerides\;\left(\mathrm{mg}/\mathrm{dL}\right)\times Glucose\;\left(\mathrm{mg}/\mathrm{dL}\right)}{2}\right)$$

$$AIP={log}_{10}\left(\frac{Triglycerides\left(\mathrm{mg}/\mathrm{dL}\right)}{\mathrm{HDL}-K\;\left(\mathrm{mg}/\mathrm{dL}\right)}\right)$$

$$PNI=10\times Albumin\;\left(g/L\right)+0.005\times Lymphocyte\;count\left(10^{3}/µL\right)$$

$$ALI=\frac{\mathrm{BMI}\;\times \;\mathrm{Albumin}\;\left(g/L\right)}{\mathrm{NLR}}$$

These indices were used to evaluate—within a multidimensional framework—the impact of inflammatory burden, metabolic dysregulation, and nutritional status on cardiovascular mortality.

### 2.5. Statistical Analysis

All statistical analyses were performed using SPSS version 25.0 (IBM Corp., Armonk, NY, USA). Continuous variables were summarized as mean ± standard deviation (SD), and categorical variables as counts and percentages (%). The distribution of continuous variables was assessed using the Shapiro–Wilk test. For non-normally distributed variables, the Mann–Whitney U test was used, whereas the independent-samples *t*-test was applied for normally distributed variables. Differences between categorical variables were analyzed using the chi-square (χ^2^) test or z-test, as appropriate.

In addition, differences between patients with and without a prior percutaneous coronary intervention (PCI), as well as between mortality groups, were evaluated separately. Statistical significance was set at *p* < 0.05. Furthermore, receiver operating characteristic (ROC) curve analysis was performed to identify variables with the highest discriminatory power for predicting mortality, and the area under the curve (AUC) was calculated.

Univariate statistical analyses were conducted to describe baseline group differences and to provide clinical context. However, inclusion of variables in the machine learning models was not restricted based on univariate statistical significance. Since machine learning algorithms are capable of capturing nonlinear relationships and interaction effects that may not be evident through conventional hypothesis-driven testing, all clinically relevant variables were retained for model training.

### 2.6. Classification

The classification process was based on multidimensional clinical, laboratory, and procedural data obtained from STEMI patients, with the aim of developing predictive models for long-term mortality.

Five supervised ML algorithms were implemented: LR, RF, XGBoost, SVM, and ANN. The dataset was partitioned into 70% training and 30% independent test sets using stratified sampling to preserve the original class distribution across both subsets.

The dataset exhibited significant class imbalance, with mortality cases representing approximately 11% of the total population. To address this, the Synthetic Minority Over-sampling Technique (SMOTE) was applied exclusively to the training set prior to model fitting, generating synthetic samples for the minority class to achieve balanced class distribution. Importantly, SMOTE was performed only within the training partition at each iteration to prevent data leakage into the test set. Additionally, algorithms supporting imbalance handling (LR, RF, and SVM) were configured with balanced class weights to further penalize misclassification of the minority class and improve sensitivity.

To reduce the risk of performance overestimation associated with a single train–test split, model robustness was evaluated using Repeated Stratified 5-Fold Cross-Validation (5 folds × 10 repetitions = 50 iterations) applied exclusively to the training dataset. Performance metrics were averaged across folds, and standard deviations were calculated to assess stability. Final model performance metrics were subsequently evaluated on the independent hold-out test set.

Continuous variables were standardized using z-score normalization prior to model training. Missing values were handled using median imputation. Hyperparameter configurations were predefined based on prior literature and empirical testing within the training dataset.

Model performance was assessed using accuracy, sensitivity, specificity, precision, F1-score, and AUC-ROC. SHAP analysis was applied to the best-performing model to quantify the contribution of each variable to mortality prediction and enhance clinical interpretability.

All input features used for machine learning model training were derived from the variables presented in Table 1, Table 2, Table 3 and Table 4, including demographic characteristics, clinical parameters, procedural variables, laboratory measurements, and calculated inflammatory–metabolic indices. No additional or hidden variables beyond those explicitly reported in these tables were included in the modeling process.

## 3. Results

In this study, classification analyses were performed using comprehensive demographic, clinical, laboratory, and angiographic data obtained from patients followed with a diagnosis of STEMI. The primary aim was to accurately and reliably predict long-term mortality risk using this multidimensional dataset. No feature selection was applied during the modeling process; instead, all available variables were directly included in the classification algorithms. This approach was intended to allow the models to utilize all potential sources of information and to avoid excluding variables that may be clinically meaningful in decision-support systems.

### 3.1. Demographic and Clinical Characteristics

Among the 329 STEMI patients included in the study, during a mean follow-up of approximately 650 days, 292 patients (88.8%) survived (survival group), whereas 37 patients (11.2%) died and were classified into the mortality (non-survival) group. The mean age of the cohort was 62.6 ± 12.1 years, and the sex distribution was 79.9% male and 20.1% female.

BMI was significantly lower in the mortality group (21.5 ± 2.1 kg/m^2^ vs. 27.8 ± 4.8 kg/m^2^, *p* < 0.001). In addition, the prevalence of COPD was markedly higher in the mortality group (75.7% vs. 8.9%, *p* < 0.001). No statistically significant differences were observed between groups with respect to other comorbidities, including DM, HT, CKD and AF. The demographic and clinical characteristics of the study population are presented in Table 1.

**Table 1:** Demographic Characteristics and Comorbidities of the Patients.

Variable	Total (*n* = 329)	Survival (*n* = 292)	Non-Survival (*n* = 37)	*p*-Value	Significance
Age (years)	62.57 ± 12.12	62.32 ± 12.20	64.54 ± 11.47	0.3690	ns
Sex				0.9731	ns
Male	263 (79.9%)	234 (80.1%)	29 (78.4%)		
Female	66 (20.1%)	58 (19.9%)	8 (21.6%)		
BMI (kg/m^2^)	27.12 ± 5.03	27.83 ± 4.85	21.54 ± 2.09	<0.001	***
DM				0.3727	ns
Yes	142 (43.2%)	123 (42.1%)	19 (51.4%)		
No	187 (56.8%)	169 (57.9%)	18 (48.6%)		
HT				0.7790	ns
Yes	202 (61.4%)	178 (61.0%)	24 (64.9%)		
No	127 (38.6%)	114 (39.0%)	13 (35.1%)		
COPD				<0.001	***
Yes	54 (16.4%)	26 (8.9%)	28 (75.7%)		
No	275 (83.6%)	266 (91.1%)	9 (24.3%)		
CKD				0.7120	ns
Yes	51 (15.5%)	44 (15.1%)	7 (18.9%)		
No	278 (84.5%)	248 (84.9%)	30 (81.1%)		
CVA/TIA				1.0000	ns
Yes	9 (2.7%)	8 (2.7%)	1 (2.7%)		
No	320 (97.3%)	284 (97.3%)	36 (97.3%)		
AF				0.7280	ns
Yes	7 (2.1%)	7 (2.4%)	0 (0.0%)		
No	322 (97.9%)	285 (97.6%)	37 (100.0%)		

Note: Values are presented as mean ± SD or n (%). ns: not significant. ***: *p* < 0.001. Abbreviations: AF: Atrial fibrillation; BMI: Body mass index; CKD: Chronic kidney disease; COPD: Chronic obstructive pulmonary disease; CVA: Cerebrovascular accident; DM: Diabetes mellitus; HT: Hypertension; TIA: Transient ischemic attack.

### 3.2. Interventional and Echocardiographic Parameters

When clinical and procedural parameters were compared, DTBT was significantly longer in the mortality group (53.6 ± 14.6 min vs. 28.3 ± 5.1 min, *p* < 0.001). Similarly, the length of hospital stay was markedly higher in the mortality group (8.46 ± 5.32 days vs. 3.81 ± 1.06 days, *p* < 0.001).

On echocardiographic assessment, LVEF was significantly lower in the mortality group (44.7 ± 10.1% vs. 51.3 ± 8.8%, *p* = 0.002). In addition, the vascular score was also associated with mortality (1.95 ± 0.78 vs. 1.56 ± 0.70 vessels, *p* = 0.004) (Table 2).

**Table 2:** Clinical and Procedural Parameters Related to Myocardial Infarction.

Variable	Total (*n* = 329)	Survival (*n* = 292)	Non-Survival (*n* = 37)	*p*-Value	Significance
Length of hospital stay (days)	4.33 ± 2.50	3.81 ± 1.06	8.46 ± 5.32	<0.001	***
DTBT (min)	31.15 ± 10.52	28.30 ± 5.07	53.65 ± 14.64	<0.001	***
LVEF (%)	50.60 ± 9.24	51.34 ± 8.86	44.76 ± 10.17	0.0002	***
Vascular score				0.0042	**
Score 1	172 (52.3%)	159 (54.5%)	13 (35.1%)		
Score 2	117 (35.6%)	104 (35.6%)	13 (35.1%)		
Score 3	40 (12.1%)	29 (9.9%)	11 (29.8%)		

Note: Values are presented as mean ± SD or n (%)., ** *p* < 0.01, *** *p* < 0.001. Abbreviations: DTBT: Door-to-balloon time; LVEF: Left ventricular ejection fraction.

### 3.3. Laboratory Findings

When laboratory parameters were evaluated, the mortality group exhibited higher WBC levels and lower HGB levels compared with the survival group (*p* < 0.05 and *p* < 0.01, respectively). Regarding inflammatory and cardiac biomarkers, CRP, troponin-I, and AST values were significantly elevated in the mortality group (*p* < 0.05). In the lipid profile assessment, HDL-C levels were lower, whereas TG levels were higher in the mortality group. Collectively, these findings support a strong association between inflammatory/metabolic stress and mortality in STEMI patients. Comparisons of laboratory parameters between groups are presented in Table 3.

**Table 3:** Laboratory Parameters. A. Complete Blood Count. B. Metabolic and Biochemical Profile. C. Lipid Profile.

Parameter	Total (*n* = 329)	Survival (*n* = 292)	Non-Survival (*n* = 37)	*p*-Value	Significance
WBC (10^3^/µL)	11.29 ± 3.55	11.21 ± 3.40	11.89 ± 4.57	0.6191	ns
HGB (g/dL)	14.28 ± 2.00	14.40 ± 1.90	13.33 ± 2.49	0.0062	**
PLT (10^3^/µL)	247.37 ± 74.85	248.32 ± 73.67	239.95 ± 84.40	0.4318	ns
Neutrophils (10^3^/µL)	7.27 ± 3.27	7.19 ± 3.11	7.92 ± 4.34	0.2651	ns
Lymphocytes (10^3^/µL)	2.15 ± 0.88	2.17 ± 0.83	2.00 ± 1.19	0.0632	ns
Monocytes (10^3^/µL)	0.76 ± 0.31	0.74 ± 0.31	0.91 ± 0.28	0.0022	**
Glucose (mg/dL)	169.63 ± 78.86	166.13 ± 77.17	197.22 ± 87.44	0.0371	*
BUN (mg/dL)	36.74 ± 14.69	35.92 ± 13.17	43.21 ± 22.73	0.1750	ns
Creatinine (mg/dL)	1.01 ± 0.49	0.99 ± 0.44	1.17 ± 0.77	0.1521	ns
GFR (mL/min/1.73 m^2^)	81.25 ± 21.09	82.21 ± 20.57	73.72 ± 23.82	0.0428	*
AST (U/L)	5.80 ± 3.15	5.73 ± 3.28	6.36 ± 1.75	0.0019	**
ALT (U/L)	48.18 ± 69.71	46.14 ± 61.68	64.27 ± 114.98	0.9407	ns
ALP (U/L)	27.96 ± 26.99	26.67 ± 19.34	38.16 ± 59.08	0.3563	ns
Uric acid (mg/dL)	77.93 ± 27.78	77.46 ± 27.53	81.65 ± 29.85	0.2709	ns
CRP at admission (mg/L)	14.78 ± 30.03	13.59 ± 28.24	24.19 ± 40.85	0.0703	ns
Peak troponin (ng/L)	18,265.74 ± 9407.72	18,571.23 ± 9113.76	15,854.84 ± 11,324.42	0.2824	ns
Albumin (g/L)	38.96 ± 4.34	39.28 ± 4.10	36.45 ± 5.37	0.0005	***
TG (mg/dL)	128.48 ± 92.12	128.45 ± 96.07	128.70 ± 52.12	0.1720	ns
Total cholesterol (mg/dL)	178.79 ± 45.38	178.68 ± 44.92	179.62 ± 49.50	0.6432	ns
HDL (mg/dL)	44.32 ± 10.56	44.63 ± 10.58	41.81 ± 10.27	0.1569	ns
LDL (mg/dL)	124.01 ± 34.47	123.54 ± 34.05	127.68 ± 37.98	0.3272	ns
HbA1c (%)	6.73 ± 1.88	6.71 ± 1.88	6.87 ± 1.85	0.7723	ns

Note: Values are presented as mean ± SD or n (%). ns: not significant. * *p* < 0.05, ** *p* < 0.01, *** *p* < 0.001. Abbreviations: ALP: Alkaline phosphatase; ALT: Alanine aminotransaminase; AST: Aspartate aminotransaminase; BUN: Blood urea nitrogen; CRP: C-reactive protein; HDL: High-density lipoprotein; HGB: Hemoglobin; GFR: Glomerular filtration rate; LDL: Low-density lipoprotein; PLT: Platelet; TG: Triglyceride; WBC: White blood cell.

Values obtained from patients’ laboratory and clinical characteristics were compared between the survivor and mortality groups, and the results are summarized in Table 4.

**Table 4:** Calculated Indices and Ratios.

Index/Ratio	Total (*n* = 329)	Survival (*n* = 292)	Non-Survival (*n* = 37)	*p*-Value	Significance
NLR	4.13 ± 3.47	3.93 ± 3.13	5.66 ± 5.24	0.0544	ns
SII	1031.21 ± 897.31	982.62 ± 792.70	1414.66 ± 1444.40	0.1082	ns
SIRI	3.18 ± 3.11	2.95 ± 2.76	5.02 ± 4.73	0.0025	**
PIV	795.14 ± 826.05	739.31 ± 748.96	1235.80 ± 1207.49	0.0096	**
TyG	9.05 ± 0.72	9.02 ± 0.74	9.28 ± 0.52	0.0095	**
AIP	0.16 ± 0.14	0.16 ± 0.15	0.15 ± 0.04	0.2062	ns
CAR	0.42 ± 0.92	0.38 ± 0.86	0.74 ± 1.28	0.0469	*
PNI	38.97 ± 4.34	39.29 ± 4.10	36.46 ± 5.37	0.0005	***
ALI	366.58 ± 216.35	380.23 ± 213.40	258.82 ± 211.93	0.0001	***

Note: Values are presented as mean ± SD or n (%). ns: not significant. * *p* < 0.05, ** *p* < 0.01, *** *p* < 0.001. Abbreviations: AIP: Atherogenic Index of Plasma; ALI: Advanced Lung Cancer Inflammation Index; CAR: C-reactive protein/Albumin Ratio; NLR: Neutrophil-to-Lymphocyte Ratio; SII: Systemic Immune-Inflammation Index; SIRI: Systemic Inflammatory Response Index; PIV: Pan-Immune-Inflammation Value; PNI: Prognostic Nutritional Index; TyG: Triglyceride–Glucose Index.

### 3.4. ML Findings

Five ML algorithms were used to predict mortality: LR, RF, XGBoost, SVM, and ANN. The dataset was randomly split into 70% training and 30% testing subsets.

#### 3.4.1. Model Performance Comparison

All models demonstrated high accuracy. The highest accuracy was achieved by XGBoost (98.9%), followed by LR (98.0%) and RF (97.9%). SVM (94.9%) and ANN (92.9%) showed comparatively lower performance.

**Figure 2:**
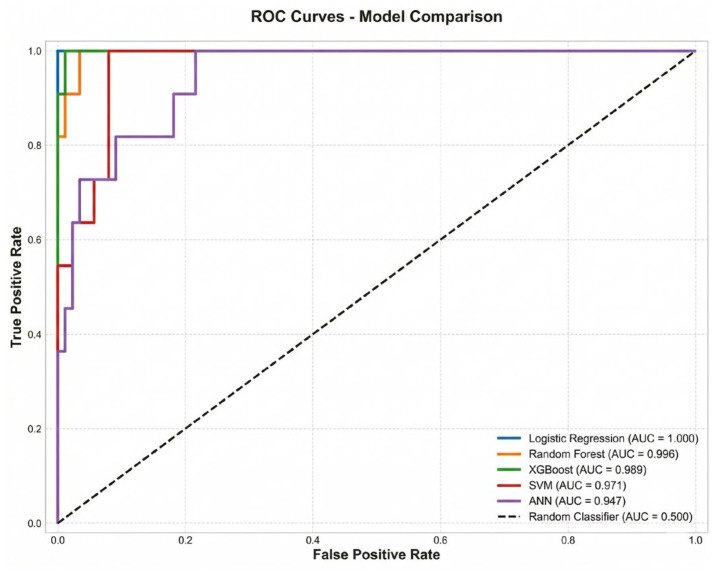
ROC curves—comparison across models.

ROC curve analysis indicated that XGBoost (AUC = 0.999) and LR (AUC = 1.000) had the highest discriminatory power (Figure 2). In both models, the AUC values were near-perfect.

**Figure 3:**
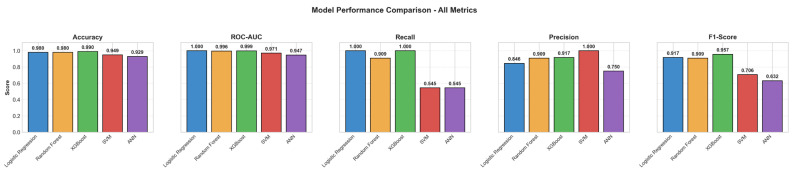
Comparison of model performance metrics (Accuracy, AUC, Recall, Precision, F1-score).

All performance metrics (Accuracy, Precision, Recall, F1-score, AUC) are summarized in detail in Figure 3 and Table 5.

**Figure 4:**
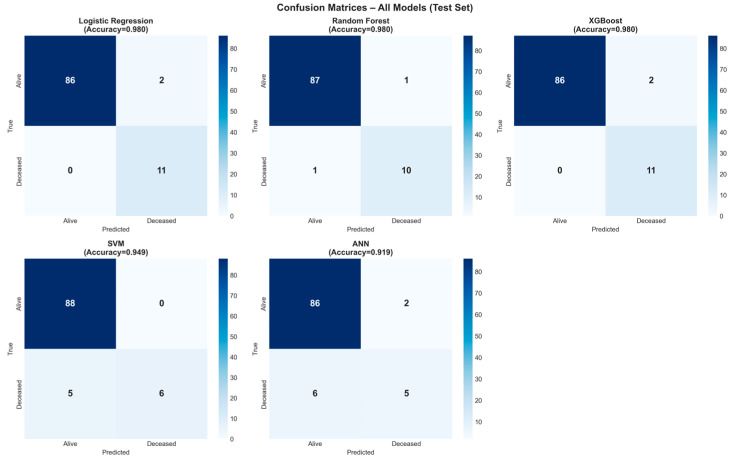
Confusion matrices of all models on the test set.

As shown in Table 5, the XGBoost model achieved the most balanced and highest scores in terms of all performance metrics. In particular, the 100% recall rate indicates that the model was able to correctly identify all mortality cases. The confusion matrices created for each model are shown in Figure 4. These matrices visually summarize the distribution of correct and incorrect classifications; it is seen that the XGBoost and LR models provide the strongest discrimination with the minimum error rate in the mortality class. In the XGBoost model, only 1 false positive case was found in the test set, and no false negative cases were detected.

#### 3.4.2. Model Calibration and Validation

**Figure 5:**
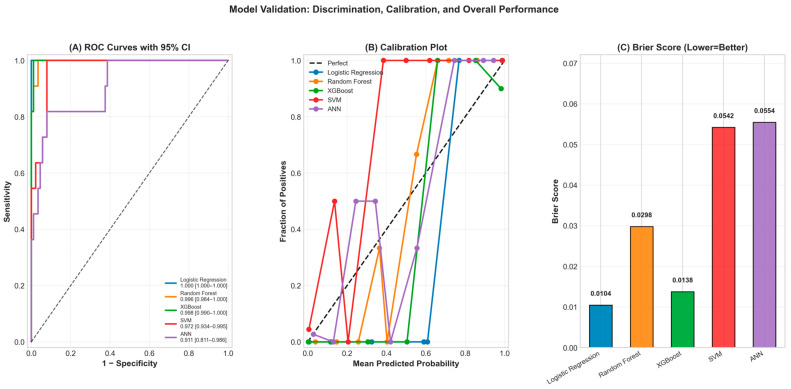
Model validation including discrimination, calibration, and overall performance metrics.

In addition to discrimination performance, model calibration and overall probabilistic accuracy were assessed for all five algorithms. Brier scores were as follows: LR = 0.0104, RF = 0.0298, XGBoost = 0.0138, SVM = 0.0542, ANN = 0.0554. All values were substantially lower than the null model reference (Brier = 0.0988), indicating that all models provided meaningful predictions beyond chance. Among these, LR and XGBoost demonstrated the strongest calibration and probabilistic accuracy. Calibration plots revealed that LR and XGBoost predictions were closely aligned with observed event probabilities across risk strata, while SVM and ANN showed greater deviation from the perfect calibration diagonal (Figure 5). The XGBoost model, which achieved the highest AUC of 0.998 (95% CI: 0.990–1.000), also demonstrated strong calibration, supporting its selection as the primary model for clinical interpretation.

**Figure 6:**
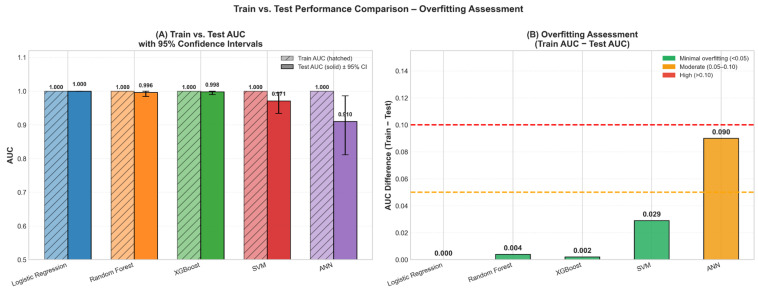
Train–test performance comparison and overfitting assessment of ML models.

To further assess potential overfitting, training and independent test set performances were compared (Figure 6, Table 6). For the XGBoost model, the training AUC was 1.000, while the test AUC was 0.998 (95% CI: 0.990–1.000), resulting in a minimal AUC difference of 0.002. Similar minimal performance gaps were observed for LR (ΔAUC = 0.000) and RF (ΔAUC = 0.004), indicating strong generalization capacity.

In contrast, larger performance discrepancies were observed for ANN (ΔAUC = 0.090), suggesting moderate overfitting. Overall, the small train–test performance gaps for the best-performing models support the robustness of the proposed approach.

#### 3.4.3. Feature Importance Analysis

To interpret the model decision mechanism and enhance clinical interpretability, feature-importance ranking and SHAP analysis were performed.

**Figure 7:**
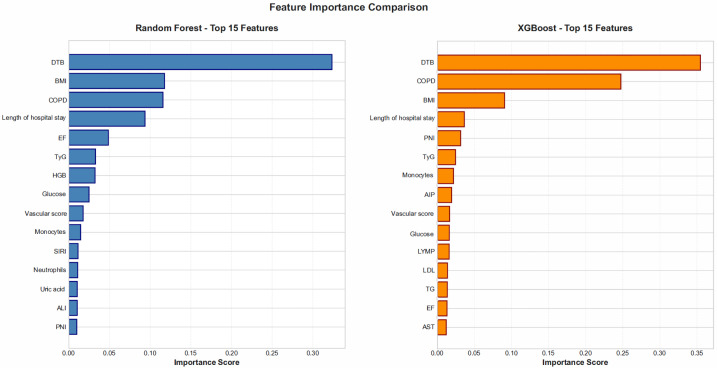
Comparative feature-importance ranking of the top 15 variables in the RF and XGBoost models.

In both the XGBoost and random forest models, DTBT emerged as the variable with the highest importance score (Figure 7). DTBT was followed by the presence of COPD, BMI, length of hospital stay, and glucose level. These findings further support the critical impact of reperfusion timing on mortality.

#### 3.4.4. SHAP Analysis

**Figure 8:**
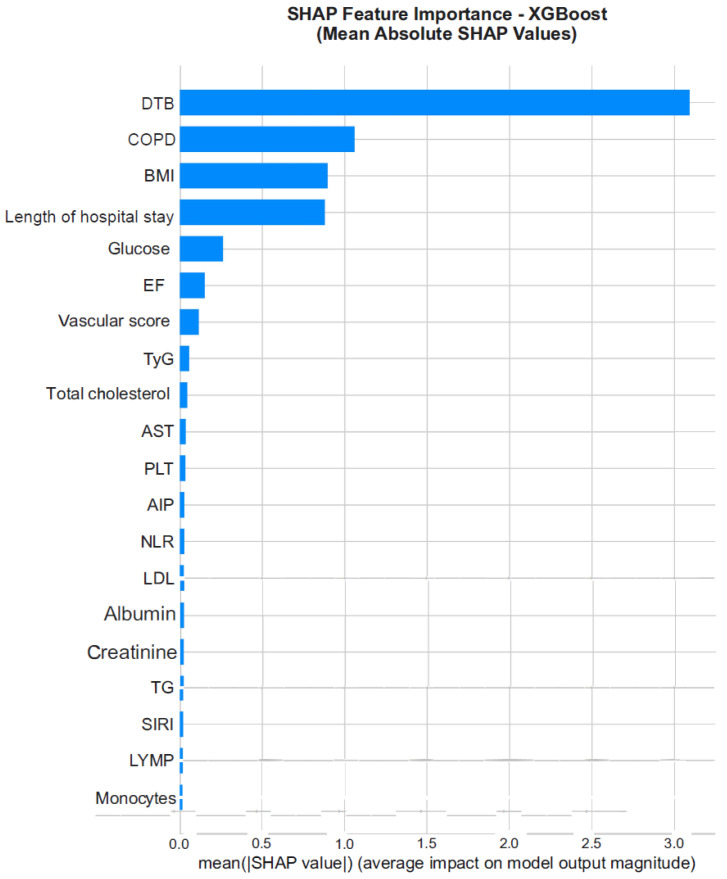
SHAP Feature Importance graph (average effects).

**Figure 9:**
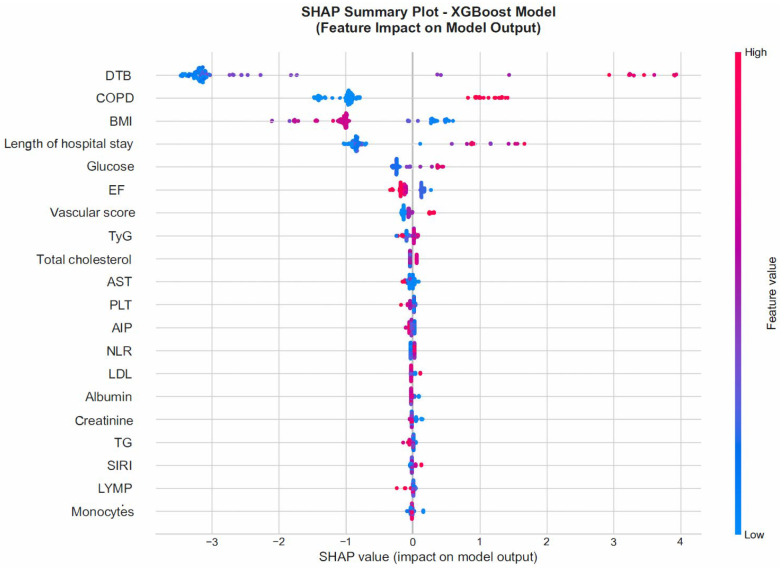
SHAP Summary Plot (direction and magnitude of feature contributions to the model output).

SHAP analysis was used to explain the decision-making process of the XGBoost model and to visualize the contribution of each feature to the model predictions. The results indicated that prolonged DTBT, presence of COPD, lower BMI, longer length of hospital stay, and reduced LVEF significantly increased the risk of mortality. In addition, higher albumin levels and lower CRP values were associated with improved survival (Figure 8 and Figure 9).

The SHAP feature-importance plot shown in Figure 6 presents the mean absolute SHAP values for each variable. The SHAP summary plot in Figure 7 visualizes both the direction and magnitude of the effect of each feature value (low to high) on the model output. In this plot, red points represent higher feature values, whereas blue points indicate lower feature values.

While feature-importance analysis ranks variables according to their relative contribution to model performance, SHAP analysis provides directionality and magnitude of each variable’s effect on individual predictions. In particular, SHAP values demonstrated how prolonged DTBT, presence of COPD, and reduced LVEF consistently shifted predictions toward higher mortality risk, whereas higher albumin and BMI values exerted protective effects. Thus, SHAP offers complementary interpretability beyond global importance ranking by quantifying the direction and individualized impact of each predictor.

#### 3.4.5. Effect Size Estimation of Key Predictors

To complement the SHAP-based feature contribution analysis with clinically interpretable effect estimates, univariate logistic regression analyses were performed for each of the top predictors identified by the XGBoost model (DTBT, COPD, BMI, LVEF, and albumin). Given the sample size constraints (*N* = 329; mortality events = 37; events per variable = 7.4), univariate rather than multivariable modeling was applied to avoid overfitting and parameter instability inherent to low EPV settings.

Prolonged DTBT was significantly associated with increased mortality risk (OR: 1.50; 95% CI: 1.37–1.89; *p* < 0.001). The presence of COPD demonstrated the strongest univariate association with mortality (OR: 31.78; 95% CI: 14.82–93.48; *p* < 0.001).

Conversely, higher BMI (OR: 0.67; 95% CI: 0.59–0.73; *p* < 0.001), higher LVEF (OR: 0.93; 95% CI: 0.89–0.97; *p* < 0.001), and higher serum albumin (OR: 0.87; 95% CI: 0.79–0.95; *p* < 0.001) were each independently associated with reduced mortality risk. These quantitative effect size estimates are consistent with the relative feature importance identified in SHAP analysis and provide complementary clinical interpretability (Table 7).

## 4. Discussion

In this study, we present a comprehensive modeling framework in which multivariable clinical, laboratory, and inflammatory indices were analyzed using ML methods to predict long-term mortality in patients with STEMI. Our findings demonstrate that both conventional risk determinants (e.g., age, LVEF, and DTBT) and next-generation inflammatory–metabolic indices (e.g., SIRI, TyG, and CAR) exert a substantial impact on mortality. The highest predictive performance was achieved with the XGBoost and LR models, both of which predicted mortality with high accuracy (98–99%). Moreover, SHAP analysis confirmed that prolonged DTBT, low serum albumin, low ALI, and a high vascular score were the parameters most strongly associated with mortality.

When the demographic characteristics of our cohort were examined, the distributions of age and sex were consistent with those reported in classical STEMI cohorts in the literature [20]. However, the significantly lower BMI observed in the mortality group (21.5 kg/m^2^) supports the concept of the “obesity paradox,” which remains controversial but has been repeatedly discussed in acute coronary syndrome (ACS) [21]. In a large-scale study by Bucholz et al. (2012), lower BMI values were associated with increased mortality among patients with ACS, and malnutrition/cachexia was suggested to reduce myocardial reserve [22]. Excessive activation of neurohormonal and inflammatory pathways may accelerate catabolic processes and impose an additional burden on myocardial metabolism. Malnourished patients may be metabolically insufficient to meet the increased myocardial energy and nutritional demands. This energy imbalance and reduced physiological reserve may represent one of the key mechanisms explaining the strong relationship between malnutrition and adverse outcomes and increased mortality in ACS [23]. In our study, the lower BMI, albumin, and PNI values in the mortality group similarly reflect the detrimental prognostic effect of malnutrition.

With respect to clinical parameters, the decisive role of DTBT on mortality aligns closely with prior evidence. Nallamothu et al. (2015) demonstrated that each 10 min delay in DTBT linearly increased mortality risk [24]. DTBT delay prolongs the duration of myocardial ischemia, thereby increasing irreversible myocyte injury [25]. Importantly, contemporary understanding emphasizes that successful myocardial perfusion is not limited to epicardial coronary artery patency; microvascular functionality also plays a pivotal role. Prolonged DTBT not only increases ischemic time but also substantially raises the risk of no-reflow and microvascular dysfunction, adversely affecting the final infarct size [26]. Consistent with this, DTBT emerged as the most influential variable in our SHAP analysis, and the mean DTBT was significantly longer in the mortality group (53.6 min; *p* < 0.001). This finding reaffirms that the timing of revascularization remains the most critical determinant of survival, independent of modern pharmacotherapy and interventional techniques.

Recent studies suggest that beyond the NLR, composite indices incorporating platelets and monocytes—such as SII and SIRI—may better reflect prognosis after STEMI [7,13]. Our results support this notion by demonstrating significantly higher levels of monocyte-based indices (SIRI) and the TyG index, which reflects metabolic dysregulation, in the mortality group. In addition, the strong association between low ALI—a parameter widely used in oncology but only recently explored in cardiology—and mortality suggests that ALI may also serve as a prognostic marker in STEMI. In contrast to some reports, we did not observe significant associations between SII, NLR, or AIP and mortality in our cohort (*p* < 0.05). This may be attributable to the limited sample size. Nevertheless, these findings indicate that in complex clinical syndromes such as STEMI—where prognosis is influenced by numerous factors—a single biomarker may be insufficient for holistic risk assessment. Accordingly, our data support the hypothesis that comprehensive and complex algorithms integrating multiple parameters may provide more reliable guidance for clinical decision-making.

AI refers to machine-based technologies capable of learning by mimicking human cognitive functions, extracting meaningful patterns from complex datasets, and generating dynamic solutions based on these data. ML, a key subfield of AI, comprises algorithms that systematically analyze data, discover latent patterns, and optimize predictive capacity as they are exposed to additional data over time [27]. This ability to “learn” and improve distinguishes ML from conventional analytical approaches. In the literature, ML-based strategies for mortality prediction in STEMI have been shown to outperform traditional risk scores such as TIMI or GRACE. Aziz et al. (2021) applied ML algorithms in 6299 STEMI patients to predict short- and long-term mortality and reported significantly higher AUC values for ML models compared with the TIMI score (0.88 vs. 0.81) [28]. In line with these findings, our ML models—particularly XGBoost and RF—demonstrated strong predictive performance beyond conventional logistic modeling. While more complex deep learning (DL) or LLM-based models are increasingly applied in large-scale or unstructured datasets, their advantages are less evident in small structured tabular datasets. Given the sample size and the nature of the available features, tree-based algorithms and classical machine learning models were considered methodologically more appropriate and less prone to overfitting.

Furthermore, Han et al. (2025) developed explainable ML models incorporating systemic inflammation indices to predict malignant ventricular arrhythmias in STEMI patients and reported an AUC of 0.925 for the RF model [13]. In our study, the AUC achieved by XGBoost was 0.999, indicating even higher discrimination. This difference may be explained by our integration of multiple indices (SII, SIRI, TyG, CAR, AIP, PIV, etc.) rather than relying on a single inflammatory marker, as well as the inclusion of clinically critical variables (DTBT, LVEF, and COPD). Collectively, this multidimensional approach may better represent biological heterogeneity.

In another relevant study, Fedai et al. (2024) used the CALLY index to predict the no-reflow phenomenon in STEMI and demonstrated that the XGBoost algorithm achieved high accuracy (>90%) [29]. Our work expands this perspective by combining multiple inflammatory and metabolic indices rather than focusing on a single biomarker, thereby yielding a potentially stronger model for clinical decision support.

Regarding interpretability, SHAP analyses revealed clinically meaningful drivers of mortality risk. Prolonged DTBT, presence of COPD, low BMI, longer hospital stay, and reduced LVEF were identified as the strongest factors increasing mortality risk. These results align with previous studies emphasizing the critical importance of reperfusion timing and comorbidity management in STEMI [28,30]. Additionally, protective factors identified by SHAP (e.g., higher albumin and lower CRP) support the prognostic role of inflammation, consistent with prior evidence highlighting the value of inflammatory biomarkers in STEMI prognosis [13].

This study has several limitations. First, it was conducted using a single-center retrospective design, which may introduce selection bias and limit the generalizability of the findings. Although our ML models demonstrated excellent performance in the internal dataset, they were not externally validated; therefore, testing in independent populations is required. Second, the predictors included in the model were limited to routinely available clinical and laboratory data. More comprehensive hemodynamic measurements, advanced imaging parameters (e.g., coronary flow reserve, microvascular perfusion indices), and richer long-term follow-up data were not available and were therefore excluded. Third, because reperfusion strategies, stent types, and pharmacological treatments (e.g., antiplatelet and statin dosing) were not uniformly documented across all cases, these factors may represent potential confounders that could influence model performance. Despite these limitations, integrating prognostic markers—whose utility in STEMI is supported by the literature—with AI/ML technologies holds substantial promise. We believe that combining these data with large-scale electronic health records to develop next-generation prognostic models will provide a strategic roadmap for future research and strengthen clinical decision-support systems. Additionally, more complex DL architectures were not extensively explored due to the limited sample size, and future studies with larger multicenter datasets may further evaluate their potential incremental benefit.

Although all clinically relevant variables were retained for model training, explicit feature selection or dimensionality reduction techniques were not applied. Tree-based algorithms such as XGBoost and RF inherently perform embedded feature selection through their split optimization process, thereby reducing the risk of unnecessary model complexity. Nevertheless, future studies may explore sensitivity analyses with reduced feature subsets to further evaluate model parsimony and generalizability.

Despite these methodological considerations, the overall objective of the present model was to provide a clinically applicable and interpretable prognostic framework rather than to maximize algorithmic complexity.

From a clinical perspective, the proposed ML model may be implemented as a bedside or web-based risk calculator using routinely available admission parameters. Integration into electronic health record systems could allow automatic risk estimation immediately after pPCI, facilitating early identification of high-risk patients who may benefit from closer monitoring or intensified therapeutic strategies. Although conventional risk scores such as TIMI and GRACE remain widely used in STEMI management, a direct comparison within the same dataset was not performed. Future studies should evaluate the incremental predictive value of ML-based approaches relative to established risk scores.

## 5. Conclusions

In this study, we demonstrated the effectiveness of ML algorithms and next-generation inflammatory–metabolic indices in predicting long-term mortality among patients with STEMI undergoing pPCI.

Based on our findings, the main conclusions are as follows:

The power of AI: Among the developed models, the XGBoost algorithm showed the most clinically robust performance by correctly identifying all mortality cases (100% sensitivity/recall). This suggests that AI-based systems may serve as strong clinical decision-support tools for the early identification of high-risk patients in emergency settings.

The importance of time: DTBT emerged as the most decisive factor associated with mortality. This finding further reinforces the vital importance of rapid revascularization in STEMI management.

Novel biomarkers: In addition to low albumin and low ALI, higher TyG and PIV values were strongly associated with poor prognosis. Because these indices can be easily derived from routine blood tests, they may be incorporated into risk stratification without additional cost.

The obesity paradox: The significantly lower BMI observed in the mortality group highlights the potential role of malnutrition and frailty as important risk factors in STEMI patients.

Overall, this multi-parametric approach—integrating clinical variables, biomarkers, and ML algorithms—may facilitate the development of personalized treatment strategies and contribute to improved survival among high-risk STEMI patients.

## Figures and Tables

**Table 5:** Comparison of performance metrics of machine learning models.

Model	Accuracy	ROC-AUC	Recall	Precision	F1-Score
Logistic Regression	0.9798	1.0000	1.0000	0.8462	0.9167
Random Forest	0.9798	0.9959	0.9091	0.9091	0.9091
XGBoost	0.9899	0.9990	1.0000	0.9167	0.9565
SVM	0.9495	0.9711	0.5455	1.0000	0.7059
ANN	0.9293	0.9473	0.5455	0.7500	0.6316

Abbreviations: ANN: Artificial Neural Networks; SVM: Support Vector Machines; XGBoost: Extreme Gradient Boosting.

**Table 6:** Training and Test Set Performance Comparison.

Model	Training	Test Set Performance					Overfitting
	AUC	AUC	95% CI	Sensitivity	Specificity	Accuracy	ΔAUC
LR	1.000	1.000	1.000–1.000	1.000	0.977	0.980	0.000
RF	1.000	0.996	0.984–1.000	0.909	0.989	0.980	0.004
XGBoost	1.000	0.998	0.990–1.000	1.000	0.977	0.980	0.002
SVM	1.000	0.971	0.934–0.995	0.545	1.000	0.949	0.029
ANN	1.000	0.910	0.811–0.986	0.455	0.977	0.919	0.090

**Table 7:** Univariate Logistic Regression Analysis of Key Predictors of Mortality.

Variable		Odds Ratio	95% CI	*p*-Value
DTBT	Door-to-Balloon Time	1.50	1.37–1.89	<0.001
COPD	Chronic Obstructive Pulmonary Disease	31.78	14.82–93.48	<0.001
BMI	Body Mass Index	0.67	0.59–0.73	<0.001
LVEF	Left Ventricular Ejection Fraction	0.93	0.89–0.97	<0.001
Albumin	Serum Albumin	0.87	0.79–0.95	<0.001

## Data Availability

Data are provided within the manuscript. If needed, the datasets generated and/or analyzed during the current study are available from the corresponding author upon reasonable request.
